# Epidemiology of asthma exacerbation in children before and after the COVID-19 pandemic: a retrospective study in Chengdu, China

**DOI:** 10.1186/s12887-023-04364-9

**Published:** 2023-11-22

**Authors:** Lei Zhang, Hanmin Liu, Tao Ai, Zijin Chen, Wei Tang, Shuai Hu, Jie Hu

**Affiliations:** 1 Department of Pediatric Pulmonology and Immunology, West China Second University Hospital, Sichuan University, Chengdu, China; 2 Department of Pediatric Pulmonology, Chengdu Women’s and Children’s Central Hospital, School of Medicine, University of Electronic Science and Technology of China, Chengdu, Sichuan China; 3 NHC Key Laboratory of Chronobiology, Sichuan University, Chengdu, China; 4 Key Laboratory of Birth Defects and Related Diseases of Women and Children, West China Second University Hospital, Ministry of Education, Sichuan University, Chengdu, China; 5 The Joint Laboratory for Lung Development and Related Diseases of West China Second University Hospital, Sichuan University, Chengdu, China; 6 Sichuan Birth Defects Clinical Research Center, West China Second University Hospital, Sichuan University, Chengdu, China

**Keywords:** Asthma exacerbation, Children, COVID-19, Postepidemic era

## Abstract

**Purpose:**

To examine the numbers and characteristics of children affected by asthma exacerbation in Chengdu, China, before and after the COVID-19 pandemic to inform efforts to manage childhood asthma in the post epidemic era.

**Methods:**

Data were retrospectively collected from children admitted for asthma exacerbation to Chengdu Women and Children’s Central Hospital between January 2017 and December 2022. Rates of hospitalization, ages of the affected children, comorbidities and infections, and relationships between hospitalization and seasonal or environmental factors were examined before and after the epidemic.

**Results:**

Fewer children were hospitalized for asthma exacerbation, yet more hospitalized children had severe exacerbation after the epidemic than before. Rates of hospitalization varied considerably with time of year, and the timing of peak hospitalizations differed before and after the epidemic. Only before the epidemic, rates of hospitalization for asthma exacerbation were positively correlated with humidity. Infants made up a smaller proportion of hospitalized children after the epidemic than before, with preschool children accounting for most hospitalizations after the epidemic. The proportion of children hospitalized for asthma exacerbation who also had pneumonia was significantly smaller after the epidemic than before. Conversely, the proportion of children hospitalized for asthma exacerbation who also had allergic diseases was significantly greater after the epidemic than before.

**Conclusion:**

The epidemiology of asthma exacerbation in children changed after the epidemic. Future efforts to manage the condition in the paediatric population should focus on severe asthma exacerbation, prevention and management of allergic diseases, and the influence of meteorological and environmental factors.

## What is known

The high incidence of asthma in China attracted attention during the COVID-19 pandemic. There are limited data on the impact of COVID-19 on asthma in children, and there is a lack of research on the impact of asthma exacerbation.

## What is new

The epidemiology of asthma exacerbation in children changed after the epidemic in Chengdu.

## Introduction

Asthma is a heterogeneous disease characterized by chronic airway inflammation and hyperresponsiveness that leads to repeated cough, wheezing, chest tightness and shortness of breath. It is the most common chronic respiratory disorder in children, affecting approximately 14% of children worldwide, a prevalence much higher than that among adults (7.7%) [1]. Indeed, more than one-third of adults with asthma develop the condition during childhood [1]. Asthma exacerbation is the most frequent reason for hospitalization for asthma, and it can be life-threatening in children. When left untreated, such exacerbations increase the risk of chronic obstructive pulmonary disease in adulthood [2].

Asthma remains a major problem in China, where its prevalence among children has increased by more than 50% per decade. Although the incidence of asthma exacerbation has declined slightly in recent years, the growing numbers of children with asthma continue to drive increases in the numbers of asthma-related hospitalizations and deaths among children [3]. Thus, greater efforts are needed to reduce the incidence of asthma exacerbation among children.

Partly in an effort to reduce the impact of the COVID-19 pandemic on vulnerable children, the Chinese government implemented a series of strict controls in January 2020 to reduce the spread of the causative virus, SARS-CoV-2 [4]. Our previous work found that during the COVID-19 pandemic, the emphasis of health care policies shifted from treatment towards disease prevention and adequate treatment facilities [4]. It is unclear how the epidemic may have affected the numbers and characteristics of children affected by asthma exacerbation in China.

Chengdu is a large city in China. To inform postepidemic efforts to manage and reduce the burden of asthma in children, we conducted one of the most detailed analyses of the numbers and circumstances of hospitalizations of children for asthma exacerbation in this major Chinese city.

## Materials and methods

### Study design and setting

Data were retrospectively collected from children hospitalized for asthma exacerbation in Chengdu Women and Children’s Central Hospital in Chengdu, China between January 1, 2017, and December 31, 2022. Asthma exacerbation and severe asthma exacerbation were diagnosed according to published criteria [5]. This study was approved by the Ethics Committee of Chengdu Women and Children’s Central Hospital [approval B2021(5)]. Consent was obtained from the legal guardians of the minors prior to study commencement. We affirm that this study complies with the Declaration of Helsinki and all patient data were kept confidential.

Data for the period from January 1, 2017, to December 31, 2019, were defined as “pre-epidemic”, while data for the period from January 1, 2020, to December 31, 2022, were defined as “post-epidemic”. In some analyses, children were stratified into the following age groups, in accordance with the criteria used to diagnose asthma exacerbation [5]: infants, < 3 year; preschool children, ≥ 3–6 year; and school children, ≥ 6–14 year. The data were collected from the Hospital Information System, Laboratory Information System, electronic medical records, Nursing Information System and other databases of Chengdu Women and Children’s Central Hospital. Data on air quality, temperature and humidity during the observation period were extracted from the public database of the Chengdu Meteorological Bureau (https://aqicn.org/city/chengdu/).

### Statistical analysis

Data were analysed statistically using SPSS 25.0 (IBM, Chicago, IL, USA). Normally distributed continuous data are expressed as the means ± standard deviations, while skewed continuous data are expressed as the medians (interquartile ranges). Data are expressed as n (%), and differences between percentages were assessed for significance using the chi-squared test. Pearson correlation analysis was used to explore potential associations of asthma with the air quality index, temperature and humidity. All statistical tests were two-sided, and results with a two-sided *P* < 0.05 were considered statistically significant.

## Results

**Fig. 1:**
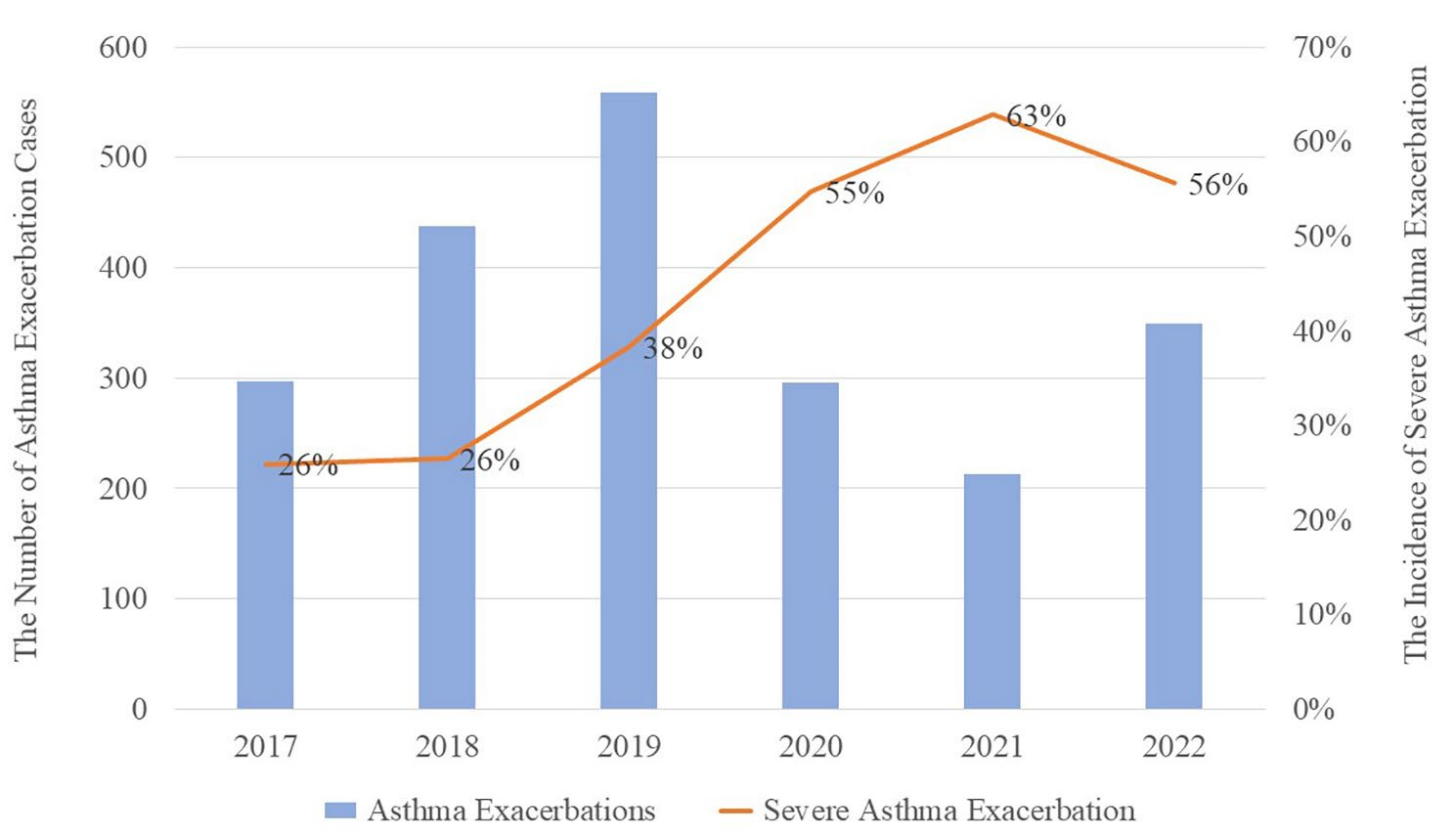
Annual number of hospitalizations for asthma exacerbation among children (blue bars, left axis) and the proportions of cases that were severe (gold line, right axis), 2017–2022

Our analysis involved 2,152 patients during the entire observation period, of whom 1,451 were male and who had a mean age of 3.17 ± 2.26 year (range, 6 mo to 14 year). The total number of children hospitalized for asthma exacerbation was 297, 438 and 559 during the three preepidemic years and 296, 213 and 349 during the three postepidemic years (Fig. 1). While the number of hospitalizations for asthma exacerbation was lower after the epidemic, the incidence of severe asthma exacerbation was higher (χ^2^ = 128.295, *P* < 0.001).

**Fig. 2:**
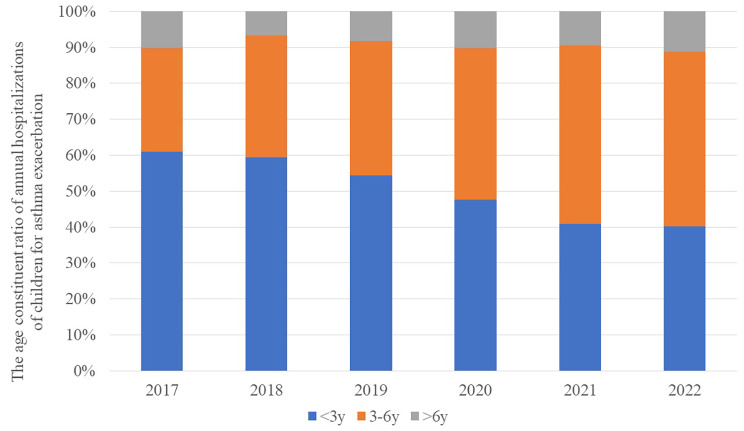
Annual proportions of hospitalizations for asthma exacerbation among children that involved infants (blue), preschool children (gold) and school children (grey). Age ranges are defined in the Methods section

Before the epidemic, infants and toddlers made up the largest proportion of children hospitalized for asthma exacerbation (Fig. 2). After the epidemic, in contrast, preschoolers made up the largest proportion (χ^2^ = 53.635, *P* < 0.001).

**Fig. 3:**
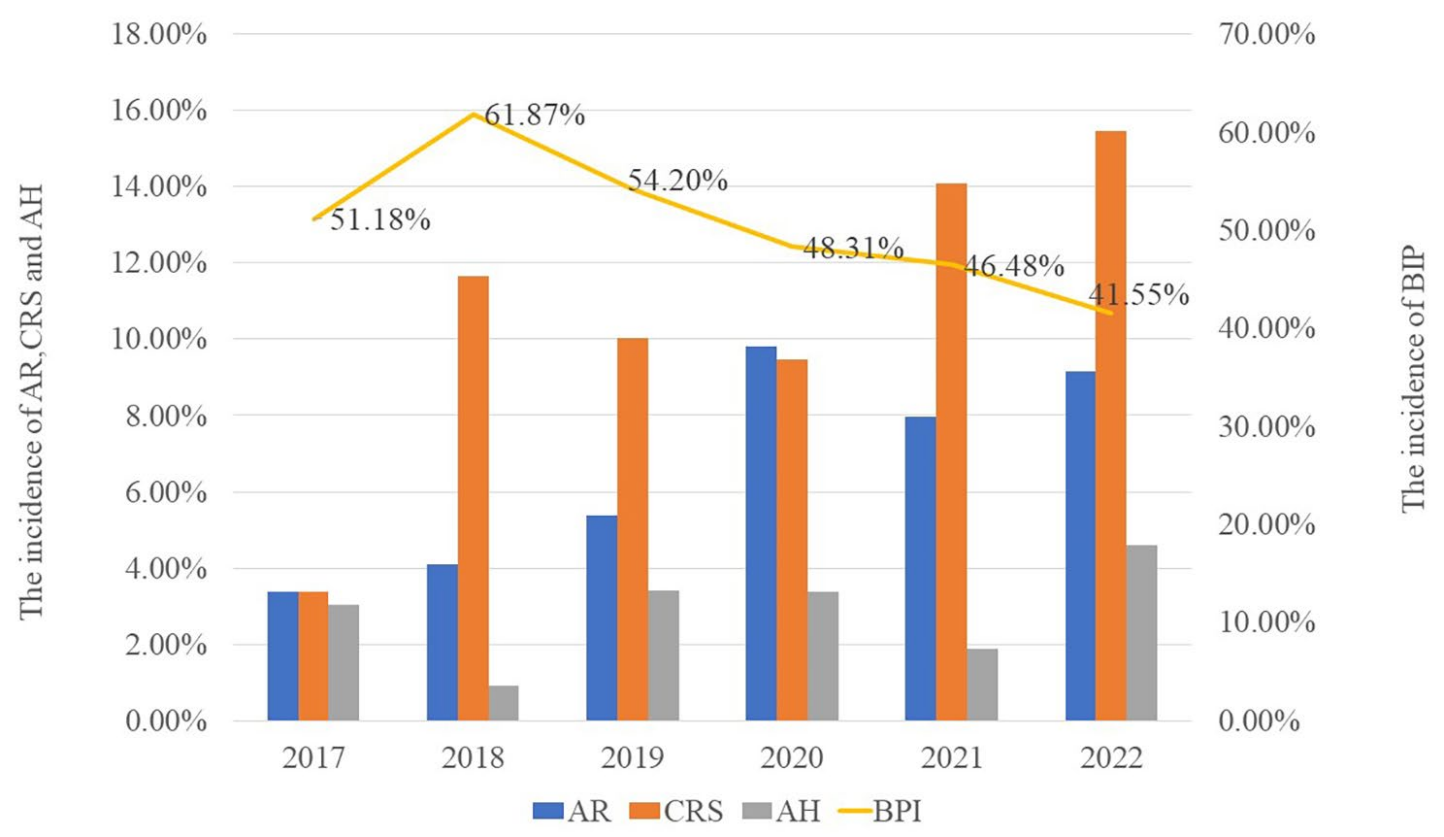
Proportions of children hospitalized annually for asthma exacerbation who also had allergic rhinitis (AR, blue bar), chronic rhinosinusitis (CRS, brown bar), adenoid hypertrophy (AH, grey bar) or bronchointerstitial pneumonia (BIP, gold line)

**Fig. 4:**
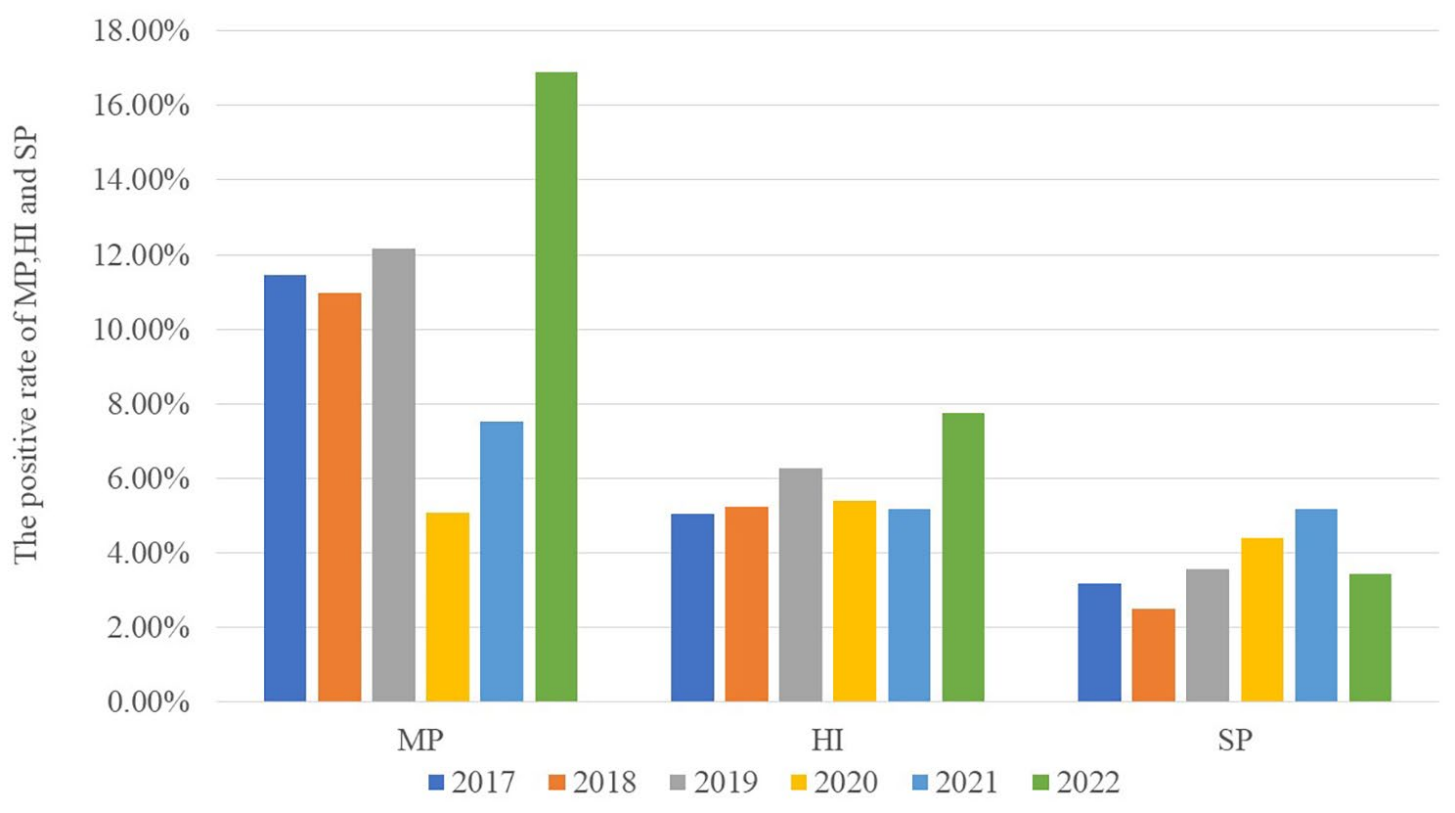
Proportions of children hospitalized annually for asthma exacerbation who were infected with *Mycoplasma pneumoniae* (MP), *Haemophilus influenzae* (HI) or *Streptococcus pneumoniae* (SP).

The proportion of children hospitalized for asthma exacerbation who also had pneumonia was significantly lower after the epidemic than before (χ^2^ = 39.948, *P* < 0.001), while the opposite was observed for allergic rhinitis (χ^2^ = 29.82, *P* < 0.001), chronic rhinosinusitis (χ^2^ = 24.579, *P* < 0.001), and adenoid hypertrophy (χ^2^ = 16.888, *P* = 0.005; Fig. 3). The proportion of hospitalized children who were infected with *M. pneumoniae* remained stable pre-epidemic (χ^2^ = 0.444, *P* = 0.801) but increased significantly postepidemic (χ^2^ = 26.31, P < 0.001). The proportions of hospitalized children who were infected with *Haemophilus influenzae* or *Streptococcus pneumoniae* remained stable pre-epidemic (χ^2^ = 3.25, *P* = 0.665) and post-epidemic (χ^2^ = 3.697, *P* = 0.594; Fig. 4).

**Fig. 5:**
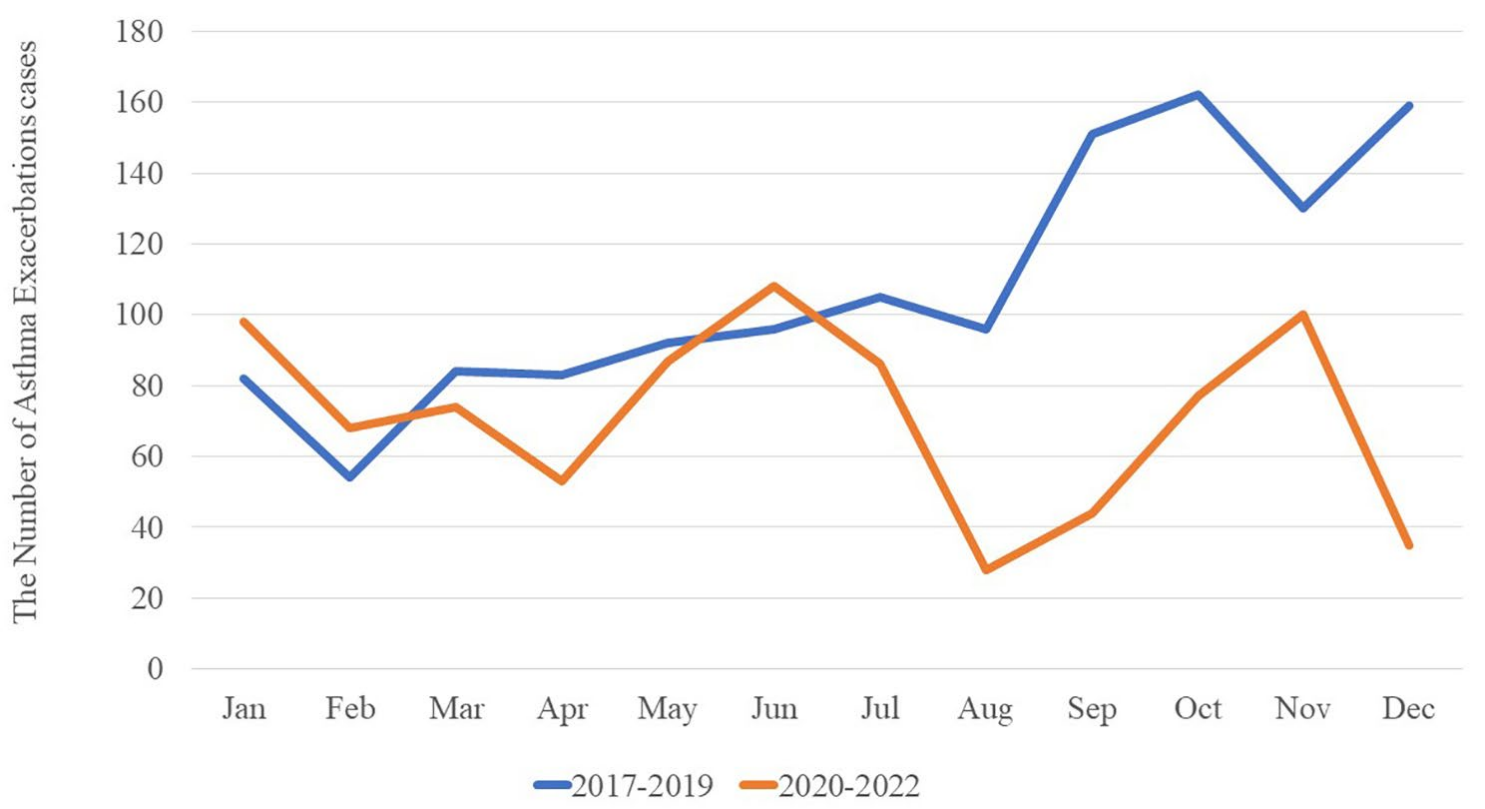
Monthly variation in the number of hospitalizations of children for asthma exacerbation during the three-year preepidemic period (blue line) and the three-year postepidemic period (gold line)

The number of children hospitalized for asthma exacerbation varied significantly by month, both pre-epidemic (χ^2^ = 1189.24, *P* < 0.001) and post-epidemic (χ^2^ = 117.01, *P* < 0.001), but the timing of the peaks differed between the two periods. One peak occurred in September-December in the preepidemic period, but separate peaks occurred in June and October in the postepidemic period (Fig. 5).

**Fig. 6:**
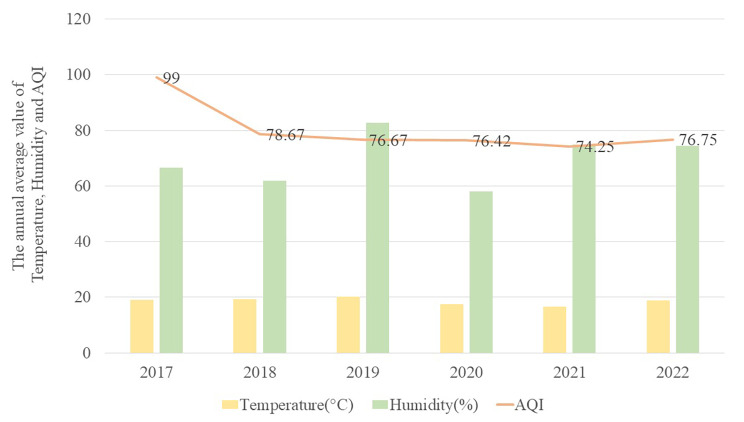
Annual average temperature (gold bars), relative humidity (percentages, green bars) and air quality index (gold line) in Chengdu during the observation period. Data were extracted from the public database of the Chengdu Meteorological Bureau

During the observation period, the average annual temperature did not vary significantly in Chengdu (*F =* 0.391, *P =* 0.853), whereas the air quality index fell continuously (*F =* 2.668, *P =* 0.029), and the average annual humidity showed peaks in 2019, 2021 and 2022 (*F* = 22.93, *P* < 0.01; Fig. 6). The number of hospitalizations of children for asthma exacerbation showed a significant positive correlation with humidity before the epidemic (*R* = 0.492, *P* = 0.015) but no correlation after the epidemic (*R* = -0.002, *P* = 0.092; Table 1). The number of hospitalizations was not significantly correlated with the air quality index or average annual temperature, either before or after the epidemic.

**Table 1:** Pearson correlation analysis of potential associations of the number of hospitalizations of children for asthma exacerbation with average annual temperature, relative humidity, and air quality index

	Temperature	Humidity	Air quality index
Pre-epidemic	-0.064 (0.767)	0.492 (0.015) *	0.061 (0.783)
Post-epidemic	-0.151 (0.482)	-0.002 (0.992)	-0.188 (0.378)

*p < 0.05 Values are correlation coefficients (associated P values)

## Discussion

Our analysis suggests that several aspects of the epidemiology of asthma exacerbation among children in China changed as a result of the COVID-19 pandemic. We found that the number of hospitalizations of children for asthma exacerbation was significantly lower after the epidemic than before. This is consistent with results reported in several countries [6–8], and it may be speculated that asthma “protects” individuals from infection with SARS-CoV-2 because the upregulation of interleukins 4 and 13 and downregulation of interferon in asthma reduce the expression of the surface protein to which the virus binds, angiotensin-converting enzyme 2 [9–11]. The decrease in hospitalizations may also reflect families’ greater concern that their children might contract infections while in the hospital, leading to greater reliance on online consultations but also greater compliance with asthma treatment [12, 13] and improved rates of asthma control [14]. In contrast to the decrease in total hospitalizations, the incidence of severe asthma exacerbations was significantly higher after the epidemic than before. This may be related to parents’ reliance on online counselling and a delay in seeking medical care. This highlights the need for greater efforts to prevent this complication and treat it in a timely manner.

Preschool children accounted for more hospitalizations post-epidemic than pre-epidemic, which may reflect their greater tendency to congregate in groups and be exposed to temperature changes than infants who have not yet entered kindergarten [15]. Our results support greater efforts to educate preschool children and teachers about asthma and public health hygiene measures, such as the use of masks and disinfectants.

The ability of strict anti-COVID-19 measures to protect against other infectious diseases likely explains why we observed a significant decrease in the proportion of hospitalizations of children with asthma exacerbation who also had pneumonia after the epidemic. On the other hand, the proportion of hospitalizations of children with asthma exacerbation who also had allergic rhinitis, sinusitis or adenoid hypertrophy increased during the observation period. Allergic rhinitis is an independent risk factor for asthma [16]. Our analysis suggests that the incidence of asthma exacerbation induced by infection decreased in the postepidemic era and that more attention should be given to the prevention and control of allergic diseases to reduce their impact on asthma.

The proportion of children who experienced asthma exacerbation and who were infected with *Mycoplasma pneumoniae* initially decreased but increased at the end of the observation period. *Mycoplasma pneumoniae* is the primary cause of community-acquired pneumonia among children in China [17, 18], and it is frequently associated with asthma exacerbation in children [19]. The observed decrease and subsequent increase may reflect initially strict COVID-19 measures in 2020 that were subsequently relaxed in 2021 and 2022. In contrast, the prevalence of infection with *Haemophilus influenzae* or *Streptococcus pneumoniae* among children hospitalized for asthma exacerbation did not change significantly during the observation period. Neither bacterial species has been linked to asthma exacerbation [20]. Our analysis suggests that anti-COVID-19 measures can be effective at reducing the spread of viruses and *Mycoplasma pneumoniae* that cause respiratory disease but not necessarily the spread of other pathogens.

Before the epidemic, hospitalizations of children for asthma exacerbation peaked from September to December, when respiratory infectious diseases are more prevalent in Chengdu [18]. This changed after the epidemic to a two-peak pattern in June and October, different from the pattern in Suzhou, China [21], which has a marine monsoon climate in contrast to Chengdu’s monsoon climate. The change in hospitalization peaks may reflect the adoption of strict hygiene measures during the epidemic, which reduced the spread of viral respiratory diseases [22]. We found that the number of hospitalizations of children for asthma exacerbation was positively correlated with relative humidity pre- but not post-epidemic, and it was not correlated with the air quality index or temperature throughout the observation period. In Chengdu, relative humidity is quite high in summer and winter, providing better living conditions for mites and mildew, among the most common allergens that affect children [23, 24]. More widespread use of air purifiers to reduce indoor dust mites and mould [25] as well as masks to prevent inhalation of allergens and viruses [26] may help reduce the influence of environmental factors on the risk of asthma exacerbation.

## Conclusion

There are limitations in this study. First, we did not analyse data on children with asthma exacerbation who were treated on an outpatient basis or only in the emergency room. In the future, the sample size should be expanded to include outpatients to draw more precise conclusions. Second, because of the imperfect data, we did not analyse in detail the prevalence of infections by common respiratory viruses among our patients. Third, the retrospective nature of our study increased the risk of bias. Finally, the temperature, relative humidity, and air quality index studied in this paper were annual mean values. If the data are daily mean values, the relationship between asthma and temperature, relative humidity, and air quality index can be further confirmed by time series analysis, which is also the direction we want to take in the next step. On the other hand, our hospital is the largest specialized children’s hospital in Chengdu, and children with asthma account for 90% of the local population, so our sample may be representative of children with asthma in the region. Nevertheless, our results should be verified and extended in large-scale prospective research.

Despite its limitations, our study is the first to investigate asthma exacerbation in children before and after the COVID-19 pandemic in this region. The findings of this study are useful for predicting asthma exacerbation in children and reducing complications caused by delayed treatment.

## Data Availability

All data generated or analysed in this study are included in this published article.

## Abbreviations

COVID-19: coronavirus disease 2019
AR: allergic rhinitis
CRS: chronic rhinosinusitis
AH: adenoid hypertrophy
BIP: bronchointerstitial pneumonia
MP: *Mycoplasma pneumoniae*
HI: *Haemophilus influenzae*
SP: *Streptococcus pneumoniae*
